# Large Fees

**Published:** 1874-09

**Authors:** 


*Large Fees.*—We take the following from a daily paper in which it appears under the head of "A Costly Dentist's Bill:"

Agnes Ethel, the actress, had cavities in four teeth filled by Dr. Atkinson, a NewYork dentist. When his bill was presented she was astonished to find that he rated his services at $1.025. She refused to pay the amount, and he brought suit against her in the New York Supreme Court. Her counsel, General Wm. H. Anthon, on applying for a bill of particulars, was furnished a bill of six items, all worded alike, except that the specific charges ranged from $50 to $300: "To services of three operators in dressing your teeth, with the gold filling necessary therefor." Judge Donohue, Tuesday, ordered that such a description of the services should be given as to enable other dentists to estimate therefrom the value of the work.

